# Effects of Azithromycin on Blood Inflammatory Gene Expression and Cytokine Production in Sarcoidosis

**DOI:** 10.1007/s00408-024-00743-w

**Published:** 2024-09-16

**Authors:** Simon D. Fraser, Susannah Thackray-Nocera, Caroline Wright, Rachel Flockton, Sally R. James, Michael G. Crooks, Paul M. Kaye, Simon P. Hart

**Affiliations:** 1 Respiratory Research Group, Hull York Medical School, Castle Hill Hospital, Cottingham, HU16 5JQ UK; 2 Respiratory Clinical Trials Unit, Hull University Teaching Hospitals NHS Trust, Castle Hill Hospital, Cottingham, HU16 5JQ UK; 3 Biosciences Technology Facility, Dept. of Biology, University of York, York, UK; 4 York Biomedical Research Institute, University of York, York, YO10 5DD UK

**Keywords:** Cough, Cytokines, Inflammation, Innate immunity, Monocyte, Sarcoidosis

## Abstract

**Introduction:**

In sarcoidosis granulomas, monocyte-derived macrophages are activated by pro-inflammatory cytokines including TNF and IL-6. Current drug treatment for sarcoidosis aims to suppress inflammation but disabling side effects can ensue. The macrolide azithromycin may be anti-inflammatory. We aimed to determine whether treatment with azithromycin affects blood inflammatory gene expression and monocyte functions in sarcoidosis.

**Methods:**

Blood samples were collected from patients with chronic pulmonary sarcoidosis enrolled in a single arm, open label clinical trial who received oral azithromycin 250 mg once daily for 3 months. Whole blood inflammatory gene expression with or without LPS stimulation was measured using a 770-mRNA panel. Phenotypic analysis and cytokine production were conducted by flow cytometry and ELISA after 24h stimulation with growth factors and TLR ligands. mTOR activity was assessed by measuring phosphorylated S6RP.

**Results:**

Differential gene expression analysis indicated a state of heightened myeloid cell activation in sarcoidosis. Compared with controls, sarcoidosis patients showed increased LPS responses for several cytokines and chemokines. Treatment with azithromycin had minimal effect on blood gene expression overall, but supervised clustering analysis identified several chemokine genes that were upregulated. At the protein level, azithromycin treatment increased LPS-stimulated TNF and unstimulated IL-8 production. No other cytokines showed significant changes following azithromycin. Blood neutrophil counts fell during azithromycin treatment whereas mononuclear cells remained stable. Azithromycin had no detectable effects on mTOR activity or activation markers.

**Conclusion:**

Blood myeloid cells are activated in sarcoidosis, but azithromycin therapy did not suppress inflammatory gene expression or cytokine production in blood.

Trial registration: EudraCT 2019-000580-24 (17 May 2019)

**Supplementary Information:**

The online version contains supplementary material available at 10.1007/s00408-024-00743-w.

## Introduction

Sarcoidosis affects an estimated 1.2 million people worldwide [1]. Many patients with sarcoidosis exhibit a chronic progressive clinical course which is associated with significant morbidity and treatment burden. There is an unmet need for new therapies for sarcoidosis that are safe, efficacious, and cost-effective. Current treatments aim to control disease by suppressing inflammatory cytokines and dampening inflammation, but at a cost of side effects which can be distressing, disabling or dangerous [2, 3].

Granulomas, the hallmark pathology of chronic sarcoidosis, are composed primarily of macrophages derived from circulating blood monocytes, activated by pro-inflammatory cytokines including TNF [4] and IL-6 [5, 6]. Sarcoid-like granulomas can be recapitulated in mice which have been genetically modified for constitutive mTOR activation in myeloid cells [7].

Azithromycin is an attractive option for repurposing for sarcoidosis. Macrolides are widely believed to exert anti-inflammatory effects distinct from anti-microbial activity [8]. Azithromycin is rapidly and highly concentrated within human monocytes [9–11]. In vitro, azithromycin promotes an anti-inflammatory macrophage phenotype [12] and suppresses mTOR activity in lymphocytes [13].

In an open label, single arm study of 21 patients with chronic pulmonary sarcoidosis and self-reported cough, treatment with 250mg azithromycin daily for 3 months reduced objective and patient-reported cough metrics. [14] Here we report the results of our analysis examining whether azithromycin treatment affects i) inflammatory gene expression and cytokine production in whole blood and ii) mononuclear cell phenotype and function and iii) mTOR activation.

## Methods

Patients with symptomatic chronic pulmonary sarcoidosis were enrolled in a noncontrolled, open label clinical trial of azithromycin 250 mg once daily for 3 months. Ethics committee approval was granted (19/YH/0100) and the trial was registered (EudraCT 2019–000580-24). Patients had a diagnosis of pulmonary sarcoidosis, > 6 months from diagnosis, and self-reported cough attributed to sarcoidosis. Patients with acute self-resolving disease were ineligible. Important exclusion criteria included bronchiectasis. Patients received oral azithromycin 250 mg once daily for 3 months. Assessments were performed at baseline (visit 1) and at 1 month (visit 2) and 3 months (visit 3) on azithromycin therapy. The study design including a description of subgroups and blood samples analyzed is shown in Fig. S1. The sample size was calculated based on cough counting which has been reported [14]. One patient dropped out after visit 1 for personal reasons. A further 3 patients did not have blood sampled at visit 3 due to the onset of the COVID pandemic.

Detail of the laboratory methods employed for inflammatory phenotyping are provided in the supplementary information.

## Results

### Inflammatory Gene Expression in Sarcoidosis Compared with Healthy Controls

Inflammatory gene expression was measured in blood samples from a subgroup of patients with sarcoidosis participating in the azithromycin trial (*n* = 8) and compared with healthy controls (*n* = 8). To minimise confounding by anti-inflammatory treatment, none of the sarcoidosis patients were taking immunosuppressant or biologic therapy. One subject was taking low dose prednisolone (5mg daily). Demographic data for the patients are shown in Table 1. All patients were white, reflecting the local population.

**Table 1:** Demographic and clinical details of patients with chronic pulmonary sarcoidosis recruited to an open label clinical trial of azithromycin for sarcoidosis cough, and the subgroup of patients analyzed for whole blood gene expression with or without ex vivo LPS stimulation using a 770-mRNA human autoimmunity panel. Continuous data are presented as median (range)

	Whole trial cohort	Gene expression subgroup
Number of subjects	21	8
Age (years)	57 (48–71)	57 (53–61)
Male/Female	9/12	1/7
Years since diagnosis	3 (1–13)	1.5 (1–11)
Scadding chest X-ray stage 1/2/3 (n)	1/7/13	1/2/5
FEV1 percent predicted	87.5 (52–131)	106 (71–131)
FVC percent predicted	91.5 (63–128)	101.5 (80–128)
FEV1/FVC ratio	0.75 (0.55–0.93)	0.77 (0.68–0.93)
Oral corticosteroid therapy (n)	4	1
Immunomodulator or biologic therapy (n)	0	0

**Fig. 1:**
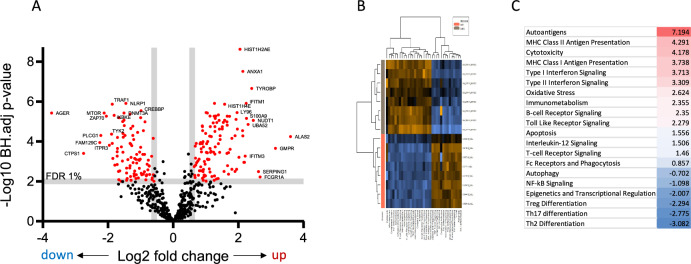
Expression of 770 inflammation-associated genes in whole blood. **A** Volcano plot comparing sarcoidosis patients (*n* = 8) and healthy controls (*n* = 8). The plot displays each gene's log2 fold change (x axis) and adjusted -log10(p-value) (y axis). Highly statistically significant genes fall at the top of the plot above the horizontal line, and highly differentially expressed genes fall to either side. The horizontal line indicates a false discovery rate (FDR) of 1%, and the vertical lines indicate fold changes of −1.5 and 1.5. **B** Heatmap of pathway scores. The plot compares pathway score changes across individual samples. Orange indicates high scores; blue indicates low scores. **C** Pathway analysis summarizing differential expression between sarcoidosis and controls. Each gene set's most differentially expressed genes are identified and the extent of differential expression in each gene set is summarized as a directed global significance score

We measured mRNA abundance in whole blood for 770 inflammation-associated genes using a targeted Nanostring nCounter® panel in the presence or absence of ex vivo stimulation for 24h with LPS. We identified 213 differentially expressed genes (DEGs; false discovery rate (FDR) < 1%, fold change (FC) > 1.5) comparing sarcoid patients to controls (118 up, 95 down; Fig. 1 and table S1). The top upregulated genes by fold change included *ALAS2* (5'-Aminolevulinate Synthase 2), *GMPR* (Guanosine Monophosphate Reductase), *FCGR1A* (Fc Gamma Receptor Ia), *SERPING1* (Serpin Family G Member 1), and *NUDT1* (Nudix Hydrolase 1). The top downregulated genes by fold change included *AGER* (Advanced Glycosylation End-Product Specific Receptor), *CTPS1* (CTP Synthase 1), *FAM129C* (Niban Apoptosis Regulator 3), *PLCG1* (Phospholipase C Gamma 1), and *MTOR* (Mechanistic Target of Rapamycin Kinase). We next used nSolver software to identify the pathways differentially regulated in sarcoidosis whole blood compared with healthy controls (Fig. 1B). Top positively regulated pathways in sarcoid patients included autoantigens (including histones), antigen presentation, cytotoxicity, interferon (type I and II) signaling, immunometabolism, and TLR signaling, indicating a general state of immune (especially myeloid cell) activation. Conversely, gene sets negatively regulated in sarcoidosis included Treg, Th17 and Th2 differentiation (Fig. 1C).

## Sarcoidosis Monocytes are Sensitive to LPS Stimulation

**Fig. 2:**
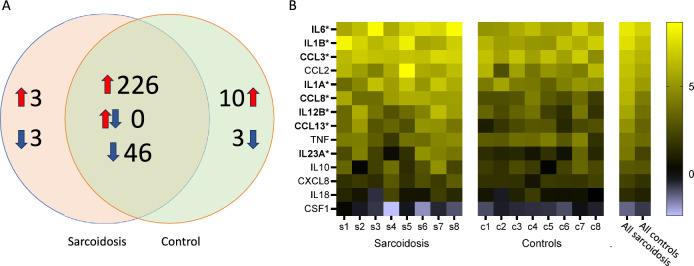
Comparison of changes in blood gene expression in response to LPS stimulation. **A** Venn diagram comparing number of genes significantly up-regulated (red arrows), down-regulated (blue arrows), or contra-regulated (both arrows) in response to stimulation with 100 ng/ml LPS for 24h between patients with sarcoidosis (*n* = 8) and healthy controls (*n* = 8). Included genes were those with at least 1.5 × fold change with LPS stimulation compared with no LPS that was statistically significant (*q* < 0.01). In addition, regulated genes in only controls or only sarcoidosis had to have measurable counts in both and at least 1.5 × fold change difference between the disease states. **B** Heatmap illustrates fold changes in cytokine and chemokine gene expression following LPS stimulation comparing blood samples from subjects with sarcoidosis (s1–s8) and healthy controls (c1–c8). On the right, columns show averaged changes in gene expression in response to LPS for subjects with sarcoidosis and controls. Scale bar represents Log2 fold changes. Gene names in bold showed statistically significant higher upregulation in response to LPS in sarcoidosis compared with controls (* *p* < 0.05, unpaired t-tests)

Monocytes are exquisitely sensitive to stimulation by LPS through binding to CD14 and TLR4, signalling through MD2 and MyD88, and activation of MAPK and NF-kB leading to production of inflammatory cytokines and chemokines including TNF and IL-6. Modulation of LPS sensitivity may thus be used to probe monocyte activation or priming. LPS stimulation led to multiple changes in gene expression compared with unstimulated blood as expected (Fig. S2). Over 95% of LPS-induced DEGs were shared between sarcoidosis patients and controls (Fig. 2A). Sarcoidosis unique upregulated DEGs in response to LPS were *FOXP1* (Forkhead Box P1), *DDIT4* (DNA Damage Inducible Transcript 4), and *IL7R*, and unique downregulated DEGs in sarcoidosis were *PYCARD* (Caspase Recruitment Domain-Containing Protein 5), *CD4*, and *PLD3* (Phospholipase D Family Member 3). Unique DEGs upregulated in response to LPS in controls included *IL27*, *TMEM176A* (Transmembrane Protein 176A), *DLL4* (Delta Like Canonical Notch Ligand 4), *CD14*, and *CD74*, and unique downregulated DEGs in controls were *ITGA4* (Integrin Subunit Alpha 4), *HIST1H3H* (H3 Clustered Histone 10), and *ID1* (Inhibitor Of DNA Binding 1). Further analysis focussing on inflammatory cytokines and chemokines showed that patients with sarcoidosis had greater fold change increases in abundance of many inflammatory mRNAs in response to LPS compared with controls (Fig. 2B).

## Effect of Azithromycin Therapy on Whole Blood Gene Expression in Sarcoidosis Patients

**Fig. 3:**
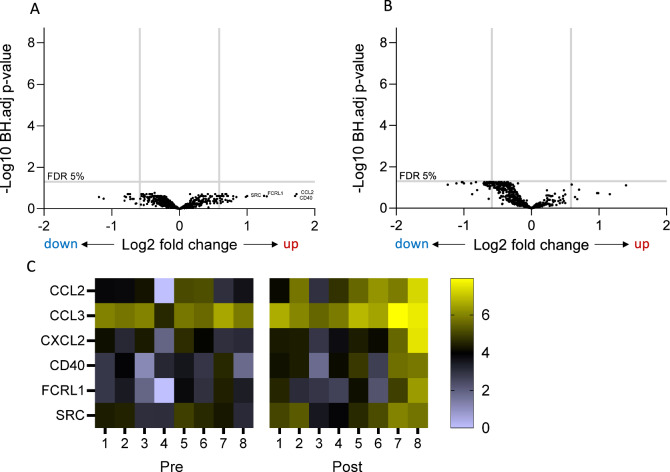
Gene expression in whole blood in sarcoidosis patients before and 1 month into azithromycin therapy. **A** Volcano plot comparing unstimulated blood pre and 1 month post azithromycin treatment. The plot shows log2 fold change (x axis) and adjusted -log10 *p*-value (y axis) comparing mRNA abundance post vs pre-treatment using paired (repeated measures) analyses (*n* = 8). The horizontal line indicates a false discovery rate of 5%, and the vertical lines indicate FC < −1.5 and > 1.5. **B** Volcano plot comparing LPS-stimulated blood pre and 1 month post azithromycin treatment. The plot shows log2 fold change (x axis) and adjusted −log10 *p*-value (y axis) comparing mRNA abundance post vs pretreatment using paired (repeated measures) analyses (*n* = 8). The horizontal line indicates a false discovery rate of 5%, and the vertical lines indicate FC < −1.5 and > 1.5. **C** Heatmap showing selected differentially upregulated genes after azithromycin therapy (without LPS stimulation). Chemokine genes (*CCL2*, *CCL3*, *CXCL2*) were identified following PLS-DA and pathway analysis with variable influence scoring. *CD40*, *FCRL1*, and *SRC* were upregulated > twofold with unadjusted *p*-values < 0.05. Gene expression is illustrated in 8 subjects with sarcoidosis before (pre) and after (post) treatment with azithromycin for 1 month. Scale bar represents Log2 normalised counts

The effect of azithromycin on whole blood gene expression was assessed in 8 sarcoidosis patients before and after 1 month of azithromycin treatment. Expression of each individual gene was analyzed in paired fashion before and after azithromycin therapy and differences presented in a volcano plot (Fig. 3). In contrast to the difference in blood transcriptome between sarcoidosis and healthy controls (Fig. 1), there was minimal effect of azithromycin therapy on whole blood gene expression, either with or without LPS stimulation (Fig. 3). No genes passed the FDR 5% and FC 1.5 threshold.

Next, we performed unsupervised and supervised clustering to give a high level exploratory view of blood gene expression before and after azithromycin therapy (Fig. S3). Unsupervised hierarchical clustering and principal component analysis did not identify clusters or variables associated with azithromycin treatment (Fig. S3 A,B). Supervised clustering with two predefined classes (pre and post azithromycin) using orthogonal partial least squares discriminant analysis (PLS-DA) and variable influence on projection (VIP) scores highlighted chemokines as the top regulated feature following azithromycin therapy (Fig. S3 C,D). Several chemokine genes showed > 1.5-fold significant upregulation following azithromycin therapy (unadjusted p values < 0.05), including *CCL2* (fold change (FC) 3.32, *p* = 0.004), *CCL3* (FC 1.6, *p* = 0.002), and *CXCL2* (FC 2.45, *p* = 0.032). The normalized counts for these mRNAs in individual subjects are shown in Fig. 3C. In addition to *CCL2*, three further genes showed > twofold upregulation in mRNA abundance following azithromycin therapy—*CD40* (*p *= 0.019), *FCRL1* (Fc Receptor Like 1, *p* = 0.009), and the protein tyrosine kinase *SRC* (*p *= 0.013).

## Effect of Azithromycin Therapy on Inflammatory Cytokine Production in Whole Blood

To determine whether azithromycin therapy has anti-inflammatory effects not reflected in whole blood gene expression profiles, we first analyzed TNF and IL-6 protein concentrations in the full azithromycin trial cohort (*n* = 21) [14]. Demographic and clinical details are shown in Table 1.

**Fig. 4:**
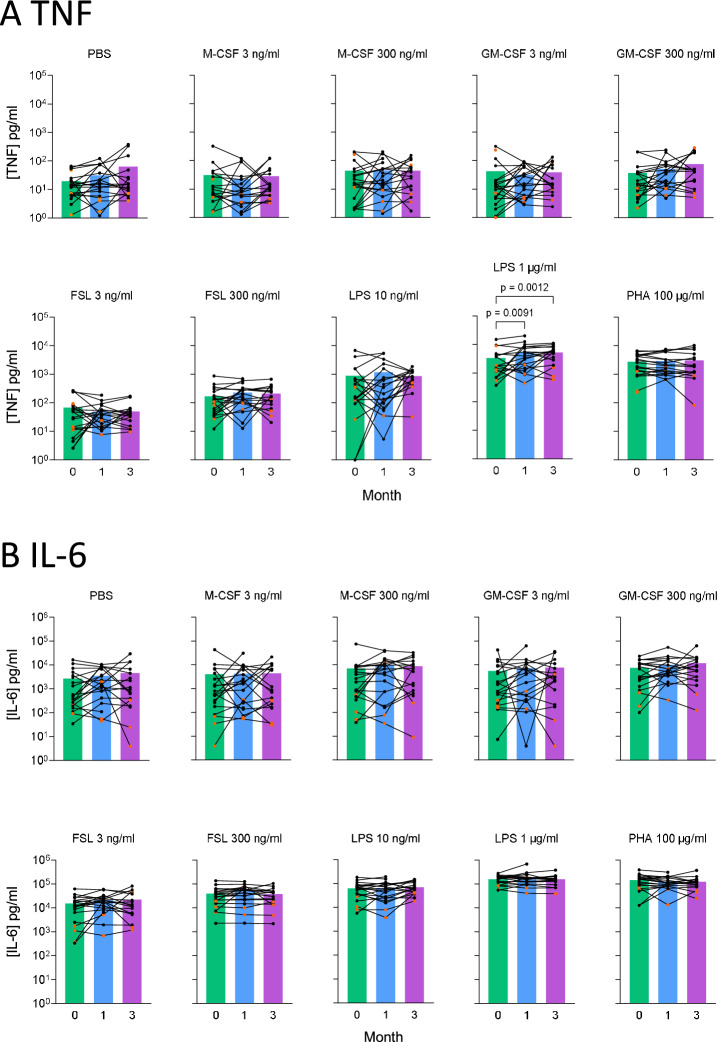
Effect of azithromycin on TNF and IL-6 concentrations in response to ex vivo stimulation of whole blood. **A** TNF and **B** IL-6 were measured in supernatants from whole blood stimulated ex vivo for 24h at 37°C under 10 conditions: PBS (control), M-CSF (CSF1 3ng/ml and 300ng/ml), GM-CSF (3 ng/ml and 300ng/ml), FSL1 (3 ng/ml and 300 ng/ml), lipopolysaccharide (LPS 1µg/ml and LPS 10ng/ml), or phytohemagglutinin (PHA 100 μg/ml). Individual patient data are plotted as dots and connecting lines (*n* = 21). Patients taking oral corticosteroid therapy are plotted in orange. Bars represent mean concentrations in plasma supernatants from samples taken at baseline (green) and following 1 month (blue) and 3 months (purple) of azithromycin therapy. Data were analyzed using a repeated measures linear mixed effects model. p values were corrected for multiple comparisons. Statistically significant results are shown on the plot

We stimulated blood samples with stimuli including TLR ligands and monocyte/macrophage growth factors. Incubation of whole blood ex vivo for 24 h under 10 individual stimulation conditions induced a range of changes in TNF and IL-6 concentrations (Fig. 4).

No inhibition of TNF or IL-6 production following azithromycin therapy was seen under any stimulation conditions. In contrast, treatment with azithromycin significantly increased TNF concentrations in response to 1µg/ml LPS (median 1782 pg/ml [95%CI 712–4383] at baseline to 3697 pg/ml [1969–7306] at 1 month; *p* = 0.0091: 5305 pg/ml [2860–6866] at 3 months; *p* = 0.0012). (Fig. 4).

## Multiplex Cytokine Assays

To look for an effect of azithromycin therapy on a wider range of inflammatory mediators, 13 cytokines were measured in plasma from ex vivo stimulated blood samples from a subgroup of six patients in the azithromycin trial who were not taking oral corticosteroid, immunomodulator, or biologic therapy. Compared with pre-treatment, azithromycin therapy for 3 months led to an increase in TNF production in response to M-CSF (*p* = 0.0278). Azithromycin did not influence concentrations of any of the other 13 cytokines in response to any stimulant (Fig. S4).

In addition, 13 cytokines were measured before and after 1 month of azithromycin therapy in sarcoidosis patients who underwent sampling using the TruCulture® (Myriad-RBM) system. Supernatants were analyzed following ex vivo stimulation with LPS or left unstimulated. IL-8 concentrations increased significantly in unstimulated TruCulture® samples following 1 month of azithromycin (median 128 pg/ml at 1 month vs 36.5 pg/ml at baseline, *p* < 0.0098, paired samples Wilcoxon test) (Fig. S5). No other changes in cytokine production were seen following azithromycin therapy.

## Effect of Azithromycin Therapy on Blood Counts and Lymphocyte and Monocyte Subsets

**Fig. 5:**
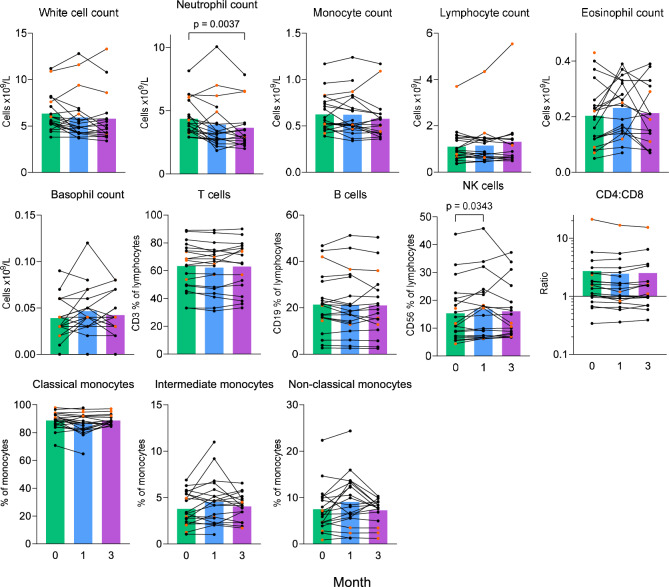
Blood cell counts and subsets in sarcoidosis patients taking azithromycin. Blood lymphocyte and monocyte subsets were assessed by expression of cell surface markers using flow cytometry. Individual patient data are plotted as dots and connecting lines (*n* = 21). Patients taking oral corticosteroid therapy are plotted in orange. Bars represent mean results in blood samples taken at baseline (green) and following 1 month (blue) and 3 months (purple) of azithromycin therapy. Data were analyzed using a linear mixed effects model. *p* values were corrected for multiple comparisons. Statistically significant results are shown on the plot

Blood neutrophil counts fell on azithromycin therapy (Fig. 5). The median blood neutrophil count was 3.98 × 10^9^/L (95%CI 3.41–4.68) at baseline, 3.2 (2.73–4.02) at 1 month (median difference −0.36 (−1.14 to −0.15), p = 0.134), and 2.98 (2.6–3.94) at 3 months (median difference −0.32 (95%CI −1.41 to -0.07), *p* = 0.0036).

There were no significant changes in lymphocyte, monocyte, eosinophil, or basophil counts. Total white blood cell counts mirrored neutrophil counts, but the fall was not statistically significant (mean difference at 3 months −0.54 × 10^9^/L (−1.21–0.13), *p* = 0.118).

No effects of azithromycin therapy on lymphocytes or monocyte subsets were seen (Fig. 5).

## Effect of Azithromycin Therapy on Cell Activation and Regulatory Markers and mTOR

Monocytes and lymphocytes were analyzed for surface activation markers CD25 and CD11b, regulatory molecules CD200L and CD200R, and intracellular mTOR activity. Azithromycin had no effect on expression of activation markers or regulatory molecules, or intracellular mTOR (mTORC1) activity as assessed by phosphorylation of S6RP (Fig.S6).

## Discussion

The blood transcriptome of patients with sarcoidosis is generally indicative of a state of heightened myeloid cell activation [15–20]. Apparent contrasts in differential expression of genes and pathways between individual studies probably reflect the different clinical and demographic features of the populations studied. Treatment with corticosteroids or non-steroid immunosuppressants is particularly likely to influence transcriptomic signatures. In our cohort patients were treatment naïve (except one who was taking low dose prednisolone), similar to cohorts described by Ascoli et al. [20] and Yoshioka et al. [19]. Other published cohorts comprised both treated and untreated patients [15, 16, 18], or treatment was not stated [17]. We focused on sarcoidosis patients with a chronic progressive clinical course since this phenotype is associated with most morbidity and treatment burden. Differences in blood transcriptomes between patients with sarcoidosis and controls reflected biologically plausible sarcoidosis-related pathways. Our finding that genes encoding several histones were upregulated in sarcoidosis is of interest since histones may be autoantibody targets in sarcoidosis [21].

Anti-inflammatory and immunosuppressive effects of azithromycin [8] have long been proposed to explain how long-term low dose therapy reduces exacerbations in patients with chronic respiratory diseases [22]. Sarcoidosis is characterised by hyper-responsiveness of monocytes in peripheral blood [23] and macrolides are concentrated within monocytes [11]. We hypothesized that treatment with azithromycin may be beneficial in chronic pulmonary sarcoidosis, and that analysis of inflammatory responses in peripheral blood would provide insights into the mechanism of azithromycin’s immunomodulatory effect.

There were minimal changes in inflammatory gene expression following treatment with azithromycin. Using a permissive FDR threshold to minimise false negatives, four genes were upregulated more than twofold following azithromycin therapy, including CCL2. Published data also show CCL2 upregulation by macrolides [24, 25]. CCL2 can prime immune cells for release of granules and secretion of pro-inflammatory cytokines [26], potentially explaining the increased cytokine responses observed in response to azithromycin without commensurate changes in cytokine gene expression.

We did not find evidence that azithromycin suppressed production of TNF, IL-6 or other cytokines in response to multiple stimuli. We showed increases in TNF and IL-8 under some conditions, suggesting that azithromycin can be pro-inflammatory. This is not the first study to report pro-inflammatory effects of macrolides. “Reversal of immune paralysis” was hypothesized to explain enhanced peripheral blood TNF and IL-6 responses in two clinical trials of macrolide therapy in patients with sepsis [27, 28]. Pro-inflammatory effects have also been reported on blood neutrophils in healthy subjects given 500mg azithromycin for 3 days [29], on IL-8 following ex vivo stimulation of whole blood with azithromycin or clarithromycin [30], on lymphocyte proliferation ex vivo [31], and on IL-6 on IL-8 production by human fibroblasts via phosphorylation of NF-κB in vitro [32]. It remains to be determined whether augmentation of cytokine responses in response to macrolide therapy is clinically meaningful, for better or worse.

Small but statistically significant falls in blood neutrophil counts were seen in patients taking azithromycin. Variable effects of macrolide therapy on blood neutrophil counts have been reported [33]. A drop in neutrophil count could be a valuable marker of adherence to therapy.

Vallet et al. described reduced T cells and altered T cell subsets in patients taking azithromycin [34]. We did not find an effect of azithromycin on lymphocyte counts or subsets. T cell lymphopenia was present in many sarcoidosis patients at baseline but was not impacted during the 3 months of the trial. Blood monocyte subsets are altered in sarcoidosis, with increased proportions of intermediate or non-classical monocytes, and fewer classical monocytes. Despite being highly concentrated within monocytes, azithromycin did not impact monocyte subsets, nor did it affect monocyte expression of the activation marker CD11b or regulatory receptor CD200R.

Our work has limitations. The relatively small sample size in the present study may have been insufficient to confirm small biological effects. Furthermore, it is possible that whole blood samples do not recapitulate local tissue inflammation in the lung. Our study was not an exhaustive analysis of immunomodulatory mechanisms. We did not study neutrophil functions directly [29, 35–37]. Azithromycin could also impact cell types not included in the blood assay, such as epithelial cells [38, 39]. Basal levels of cytokines may be derived from a variety tissues, whereas our stimulation assay will only detect changes in blood cell-derived mediators. Some cytokines may be present in granules rather than generated de novo through RNA transcription, which may explain mismatches between transcriptomic and protein data. CCL2 for example, is released from vascular endothelial cells, and moderate basal blood levels may have masked a change in CCL2 production by blood immune cells. Likewise, our assay may have missed changes in mediators with long plasma half-lives. We attempted to mitigate plateau effects, but the highest concentration of LPS and PHA used in stimulation assays may be too strong to be able to detect an inhibitory effect (9). In the absence of a placebo-treated group, increases in cytokines over time could reflect random variation or a consequence of intercurrent illness. The lack of any conditions showing a reduction in cytokine production on azithromycin therapy argues against random variation. Whilst the single arm azithromycin trial can be criticized because there was no placebo group, the repeated measures design means we expect that a meaningful immunomodulatory effect of azithromycin, if present, would have been detected with the multiple metrics that were applied to the samples.

In vitro, azithromycin blocks intracellular killing of mycobacteria [40]. It is intriguing to speculate that if, as various lines of evidence suggest, sarcoidosis is driven by microbial antigens, the increased cytokine production that we observed could reflect alterations in mycobacterial processing. However, a double-blind, placebo-controlled trial showed that 16 weeks’ treatment with azithromycin in conjunction with levofloxacin, ethambutol, and rifabutin had no effect on forced vital capacity in patients with pulmonary sarcoidosis [41].

Our findings should raise further questions on the mechanism of action of azithromycin. Indirect effects may explain anti-inflammatory endpoints in clinical trials. Reductions in inflammatory markers in response to azithromycin therapy in patients with bacterial airways colonisation (bronchiectasis, chronic obstructive pulmonary disease, cystic fibrosis) are confounded by the antibiotic action of macrolides. A strength of our study is that airways colonisation with bacteria is not a recognised feature in sarcoidosis (patients with bronchiectasis were excluded in the protocol). In lung transplant recipients, reduction in chronic rejection with macrolide therapy occurs in the context of concurrent potent immunosuppression [42, 43], with a likely explanation being an impact of azithromycin on gastrointestinal motility, hence reducing micro-aspiration events. This may also explain the benefit of azithromycin in airways disease and in some patients with chronic cough, including sarcoidosis cough [14]. Uptake of long-term azithromycin therapy is tempered by concerns about potential effects on the microbiome and antimicrobial drug resistance [44]. Non-antibiotic alternatives would be valuable. Further research is needed to understand the direct and indirect effects of macrolide therapy.

## Supplementary Information

Below is the link to the electronic supplementary material.

Supplementary file1 (PDF 2111 KB)

## Data Availability

Data are openly available through the Gene Expression Omnibus, accession GSE274707, at https://www.ncbi.nlm.nih.gov/geo/query/acc.cgi?acc = GSE274707.

## Abbreviations

FSL: Fibroblast-stimulating lipopeptide
GM-CSF: Granulocyte–macrophage colony stimulating factor
IL: Interleukin
LPS: Lipopolysaccharide
M-CSF: Macrophage colony stimulating factor
mTOR: Mechanistic target of rapamycin
PHA: Phytohemagglutinin
S6RP: Phosphorylated S6 ribosomal protein
TLR: Toll-like receptor
TNF: Tumor necrosis factor
