# Supplementary material for: Effects of Azithromycin on Blood Inflammatory Gene Expression and Cytokine Production in Sarcoidosis
Source: Lung. 2024 Sep 16;202(5):683–93. doi: 10.1007/s00408-024-00743-w (PMC11427505; doi:10.1007/s00408-024-00743-w)

SUPPLEMENTARY INFORMATION

Effects of azithromycin on blood inflammatory gene expression and cytokine production in sarcoidosis

Simon D. Fraser, Susannah Thackray-Nocera, Caroline Wright, Rachel Flockton, Sally R. James, Michael G. Crooks, Paul M. Kaye, Simon P. Hart

Contents

Methods 1

Fig.S1 Study design 4

Fig.S2 Gene expression in whole blood in sarcoidosis patients at baseline comparing LPS with no LPS stimulation 5

Fig.S3 High level exploratory view of blood gene expression comparing before and during azithromycin therapy. 6

Fig.S4 Multiplex analysis of 13 cytokine concentrations in plasma in response to ex vivo stimulation 7

Fig.S5 Multiple cytokine concentrations before and after 1 month of azithromycin therapy 8

Fig.S6 Cell activation and regulatory markers and mTOR in sarcoidosis patients taking azithromycin . 9

Table S1. Genes significantly upregulated and downregulated in sarcoidosis compared with controls 10

Appendix 1. Flow cytometry gating 13

Methods

Gene expression

A subgroup of patients (Figure 1) had blood samples collected into the TruCulture™ system (Myriad-RBM) which allows cell stimulation and sample processing with minimal handling. Blood (1ml) from patients with sarcoidosis at baseline and 1 month were drawn into tubes (TruCulture®, Myriad-RBM) containing 100 ng/ml LPS or null control (no stimulant). After stimulation for 24h at 37°C, samples were separated into supernatants (aliquoted and stored at -70°C for later cytokine analysis) and cell pellets (mixed with 3ml RNAlater® (Ambion) and stored at -70°C). RNA was extracted from 2 mls sample using the RiboPure™ RNA Purification Kit, blood (Invitrogen; Thermo Fisher Scientific). Samples were spun at 800 x g for 10 minutes, the supernatant removed, and loose blood cell pellet was carried forward into the protocol and continued according to the manufacturer's instructions. Samples were eluted from the columns into 100 µl EB. 80 µl eluted RNA was then cleaned and concentrated using RNAClean XP (Beckman Coulter); briefly, 1.8 volumes RNAClean XP solution was added to the sample, and incubated at room temperature for 10 minutes, prior to magnetic separation. The supernatant was removed, and pellet washed twice with 70 % ethanol, briefly air dried, then RNA eluted into 10 ul nuclease free water. 100 ng of the resulting RNA was then hybridised to probes from the nCounter XT autoimmune profiling panel (NS_Hs_AI_Pro_v1.1,

NanoString Technologies, 770 genes involved in 35 pathways and processes), according to the manufacturer's guidelines using an 18-hour hybridisation. Sample slides were prepared, and absolute counts generated using the nCounter FLEX analysis system. Gene expression data for healthy control samples processed with TruCulture™ and analysed using the nCounter XT human autoimmune profiling panel (NS_Hs_AI_Pro_v1.1) were provided by NanoString® as raw RCC files.

Data were normalized and analysed using nSolver 4.0 (NanoString®). Azithromycin treatment effect on gene expression was assessed using a paired analysis with visit as predictor and subject ID as confounder. False discovery rate was set at $q<0.05$. The Benjamini-Hochberg method was used to adjust for multiple comparisons. Gene expression data for healthy controls were provided by NanoString, from samples processed with TruCulture™ identically to the sarcoidosis population and analysed using the same human autoimmunity panel.

Gene set analysis (GSA) was performed using nSolver™ advanced analysis 2.0 GSA module. Gene sets were defined using NanoString NS_Hs_AI_Pro_v1.1 probe annotations. GSA summarizes change in expression within defined gene sets. A high directed global significance score (positive or negative) indicates that a large proportion of the genes in a pathway are exhibiting changes in expression (up or down) across groups of samples.

Whole blood stimulation assay

We used a whole blood assay which has advantages over methods that use isolated blood immune cells. The whole blood assay involves less cell perturbation, retains a variety of other cells and factors in blood, and is more sensitive for measuring cytokine production [23, 27]. We previously demonstrated that release of pro-inflammatory cytokines IL-6 and TNF, which are highly expressed in sarcoid-affected tissues [28], was enhanced in whole blood in response to PHA stimulation in patients with sarcoidosis compared with controls [23]. Our choice of additional stimuli for the present study was directed by the key role of monocyte-derived macrophages in sarcoidosis pathology, and the link between sarcoidosis and exposure to microbes which activate monocytes through engagement of innate immune receptors such as TLRs. Moreover, increased activity of the mTOR intracellular signalling pathway in monocytes/macrophages has emerged as a key driver of granulomatous inflammation in mouse models and patients with chronic sarcoidosis [7]. Ex vivo stimulation with growth factors M-CSF or GM-CSF, or TLR ligands FSL1 (TLR2/6) and LPS (TLR4) all activate mTOR in monocytes, mirroring the sarcoid granuloma. We chose two concentrations of most stimuli to avoid plateau effects whereby maximal cytokine production is beyond inhibition.

Stimulants were added to 2ml round bottom microfuge tubes. Stimulants were phosphate-buffered saline (PBS, control), growth factors macrophage colony stimulating factor (M-CSF, CSF1) or granulocyte-macrophage-colony stimulating factor (GM-CSF, CSF2) (both 3 ng/ml or 300 ng/ml), TLR4 ligand lipopolysaccharide (LPS; 10 ng/ml or 1 µg/ml), TLR2/6 ligand fibroblast-stimulating Lipopeptide (FSL1, Pam2CGDPKHPKSF; 3 ng/ml or 300 ng/ml), or the mitogen lectin phytohemagglutinin (PHA 100 µg/ml).

1 ml of heparinised blood was added to each tube containing stimulants at 1% of the volume. Tubes were incubated at 37°C for 16 hours without agitation or rotation. After incubation, tubes were centrifuged for 8 minutes at 10,000 rpm in a benchtop centrifuge. Plasma (400-600µl) was aspirated and transferred to labelled tubes for storage at -20°C.

Cytokine measurement

TNF and IL-6 were measured by ELISA (Biolegend) according to the manufacturer's instructions. Standard curves were calculated using Hycult AssayPro software (Hycult). Samples recorded as below the LLD of the assay (7.8 pg/ml) were imputed as half the lowest standard concentration (3.9 pg/ml).

Samples from 10 patients with sarcoidosis at visits 1 (baseline) and visit 2 (1 month during azithromycin treatment; Figure 1) were processed using TruCulture® OptiMAP technology with ex vivo stimulation with LPS 100ng/ml or control for 24h at 37°C. 13 cytokines were assayed by ELISA (Myriad RBM).

Samples from 6 steroid-naïve patients with sarcoidosis at visits 1 (baseline) and 3 (3 months azithromycin; Figure 1) were analysed for 13 cytokines using a multiplex flow cytometric assay (LEGENDplex ™ Human Inflammation Panel 1, BioLegend®). Stimuli were PBS (control), M-CSF 300ng/ml, GM-CSF 300ng/ml, FSL1 300ng/ml, LPS 1 µg/ml and 10ng/ml, and PHA. Data were processed using the LEGENDplex™ Data Analysis Software Suite (BioLegend).

Flow cytometry

Mononuclear cells were purified from heparinised blood using Histopaque®-1077 gradient centrifugation. PBMCs were stained using monoclonal antibodies via extracellular staining protocols with appropriate controls. Lymphocyte and monocyte subsets and surface markers were analysed by multi-parameter flow cytometry (13). Blood lymphocytes were analysed for CD3+ T cells, CD3+ CD4+ T cells, CD3+ CD8+ T cells, and all NK cells (CD3- CD19- CD56+). Blood monocytes were analysed for CD14++ CD16- classical monocytes, CD14++ CD16+ intermediate monocytes, and CD14+ CD16++ non-classical monocytes. Subsets were defined using dual staining with CD14 and CD16 and isotype controls to establish gates using pseudocolour dot plots. Classical monocytes were defined as those with no CD16 expression but CD14 high expression (CD14++ CD16-) and defined the remaining cells as intermediate (CD14++ CD16+) and non-classical (CD14+ CD16++). mTOR activity (specifically mTORC1) was assessed by flow cytometric measurement of intracellular phosphorylated S6RP. Isolated PBMCs were stained for CD3, CD14 and CD16 by extracellular staining as described previously, fixed using fixation buffer (Biolegend, 4 % paraformaldehyde) for 20 minutes and permeabilised with True-Phos™ Perm Buffer (Biolegend) as per the manufacturer's instructions. Samples were incubated with anti-phospho-S6RP and isotype control antibodies and analysed by flow cytometry.

Statistical analysis

Azithromycin treatment effect on gene expression was assessed using a paired analysis with visit as predictor and subject ID as confounder. False discovery rate was set at $q<0.05$. The Benjamini-Hochberg method was used to adjust for multiple comparisons. Descriptive statistics are reported as medians and ranges. Tests of normality were performed, and log transformation performed when appropriate. Changes in parameters over time were analyzed with a linear mixed effects model. In the repeated measures design, the Geisser-Greenhouse correction was applied when epsilon <0.75. A compound symmetry covariance matrix was used, and model fit was with restricted maximum likelihood. Comparison between conditions were performed using Holm-Šídák tests, with correction for multiple comparisons within each analysis based on statistical hypothesis testing. p-values were not adjusted for multiple comparisons across different analyses. Paired Wilcoxon tests were used for paired data with two time points. GraphPad Prism (version 10.0.0) was used for the analyses. A multiplicity-adjusted P value <0.05 was regarded as statistically significant.

Fig.S1 Study design

LPS, lipopolysaccharide; PHA, phytohemagglutinin; TLR, Toll-like-receptor.

[a] IL-1β, IL-6, IL-10, IL-12p70, IL-17A, IL-18, IL-23, IL-33, IFN-α2, IFN-γ, CCL2, TNF

[b] CXCL5, GM-CSF, IFN-γ, IL-1β, IL-2, IL-6, IL-8, IL-10, IL-12p70, IL-13, IL-17, IL-23, TNF

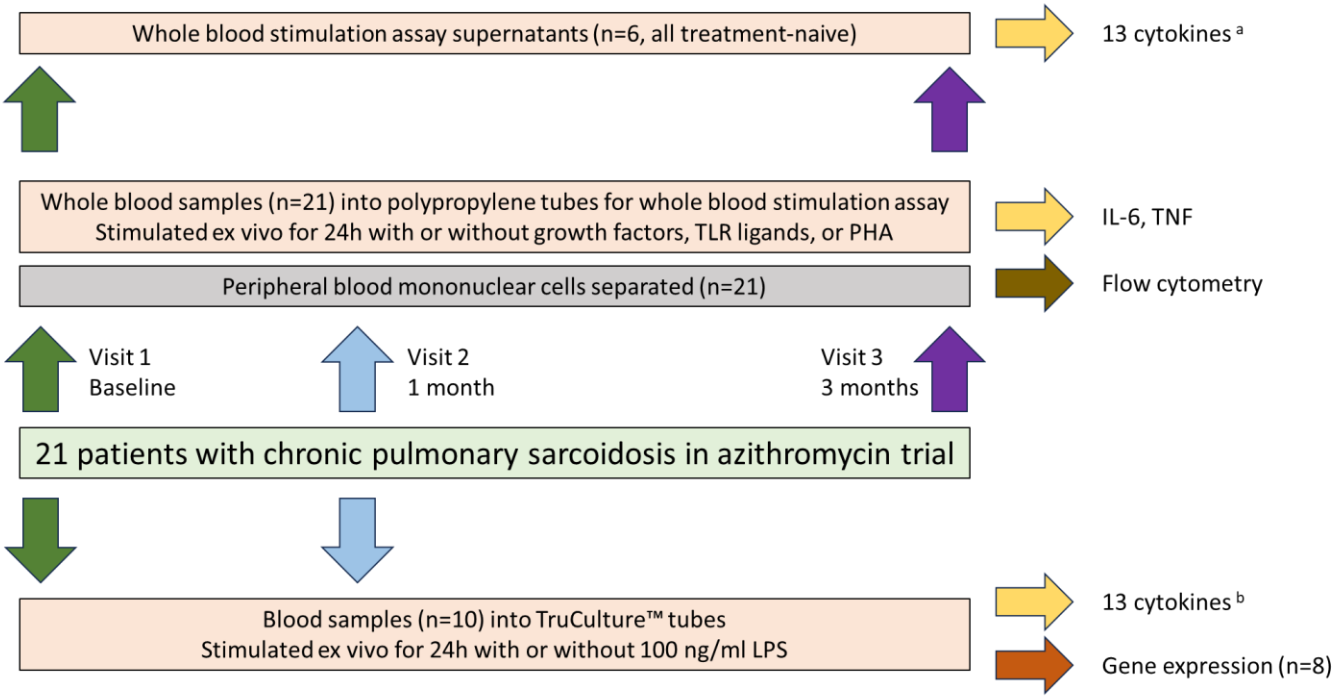

Fig.S2 Gene expression in whole blood in sarcoidosis patients at baseline comparing LPS with no LPS stimulation

Volcano plot displaying each gene's log2 fold change (x axis) and adjusted -log10(p-value) (y axis). Genes above the horizontal line have FDR <0.05. Genes either side of the vertical lines are up- (right) or down- (left) regulated >1.5-fold in response to ex vivo stimulation of whole blood with 100 ng/ml LPS for 24h at 37°C.

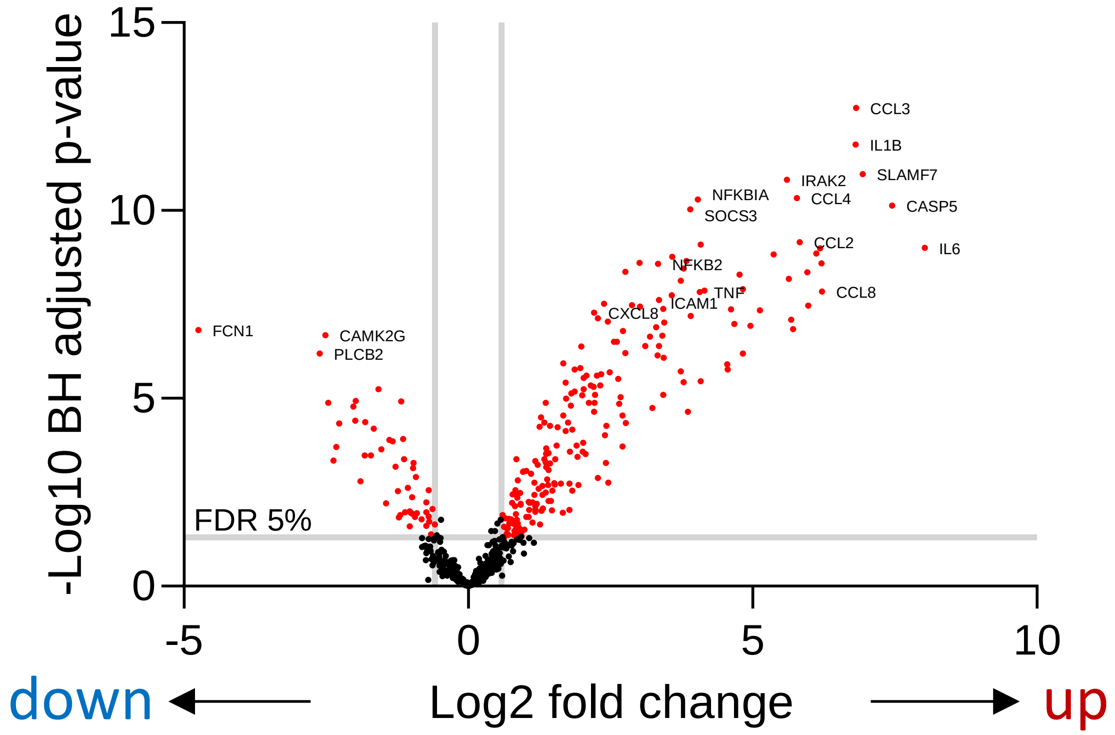

Fig.S3 High level exploratory view of blood gene expression comparing before and during azithromycin therapy.

A. Unsupervised clustering heatmap of the normalized data. Data are scaled to give all genes equal variance; orange indicates high expression; blue indicates low expression. Samples from individual subjects (columns, n=8) are generally clustered together, whereas azithromycin treatment (1, before or 2, during azithromycin therapy) is not clustered. B. Principal component analysis (PCA). Two principal components derived from the gene expression data are plotted against each other and colored by azithromycin treatment (green, before and blue, during azithromycin therapy). PCA shows overlap between samples taken pre- and post- azithromycin treatment. Unsupervised analysis using PCA does not identify clusters or variables associated with prominent signals in the data. PCA analyses performed using nSolver™ 4.0 advanced analysis package (NanoString®). C. Supervised clustering using orthogonal partial least squares discriminant analysis (OPLS-DA, SIMCA® v.18, Umetrics®) with two classes input as pre and post azithromycin therapy. By default, the observations are assigned to the nearest class. D. Biomarker analysis using BigOmics® analytics showing gene sets most reflecting azithromycin therapy based on a variable importance score for each feature. The top features are listed according to cumulative ranking.

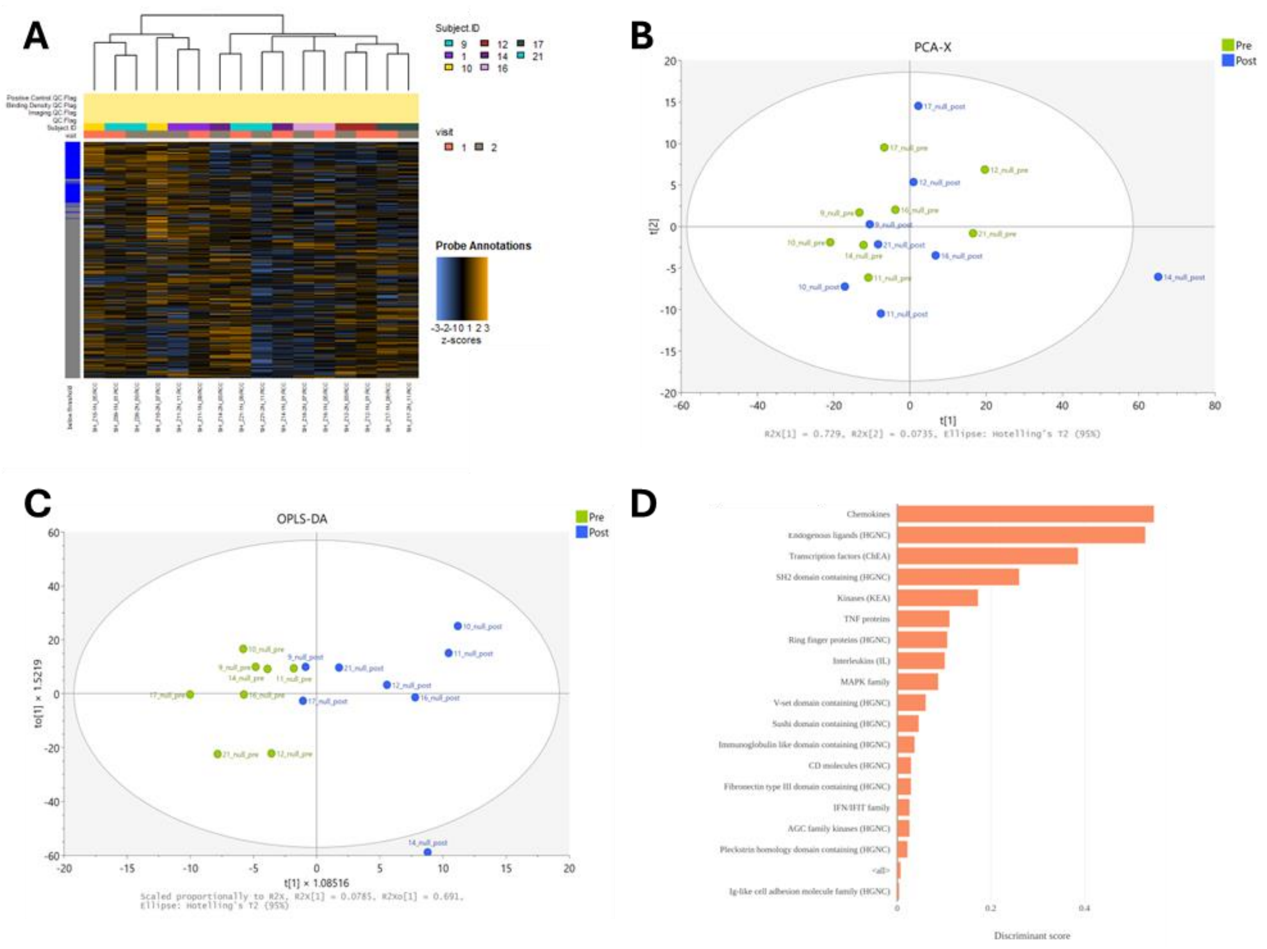

Fig.S4 Multiplex analysis of 13 cytokine concentrations in plasma in response to ex vivo stimulation

Plasma from blood stimulated ex vivo with M-CSF 300ng/ml, GM-CSF 300ng/ml, FSL1 300ng/ml, LPS 1µg/ml, LPS 10ng/ml, PHA 100µg/ml, or PBS (control) was analyzed for six patients with sarcoidosis who were not taking oral corticosteroid, immunomodulator, or biologic therapy at baseline (green) and 3 months (purple) on azithromycin therapy. Boxplots show median (horizontal line), 95% confidence interval (box), and range (whiskers). Individual patient data are plotted as dots. Data were analyzed using a linear mixed effects model. p values were corrected for multiple comparisons. Statistically significant results are shown on the plot.

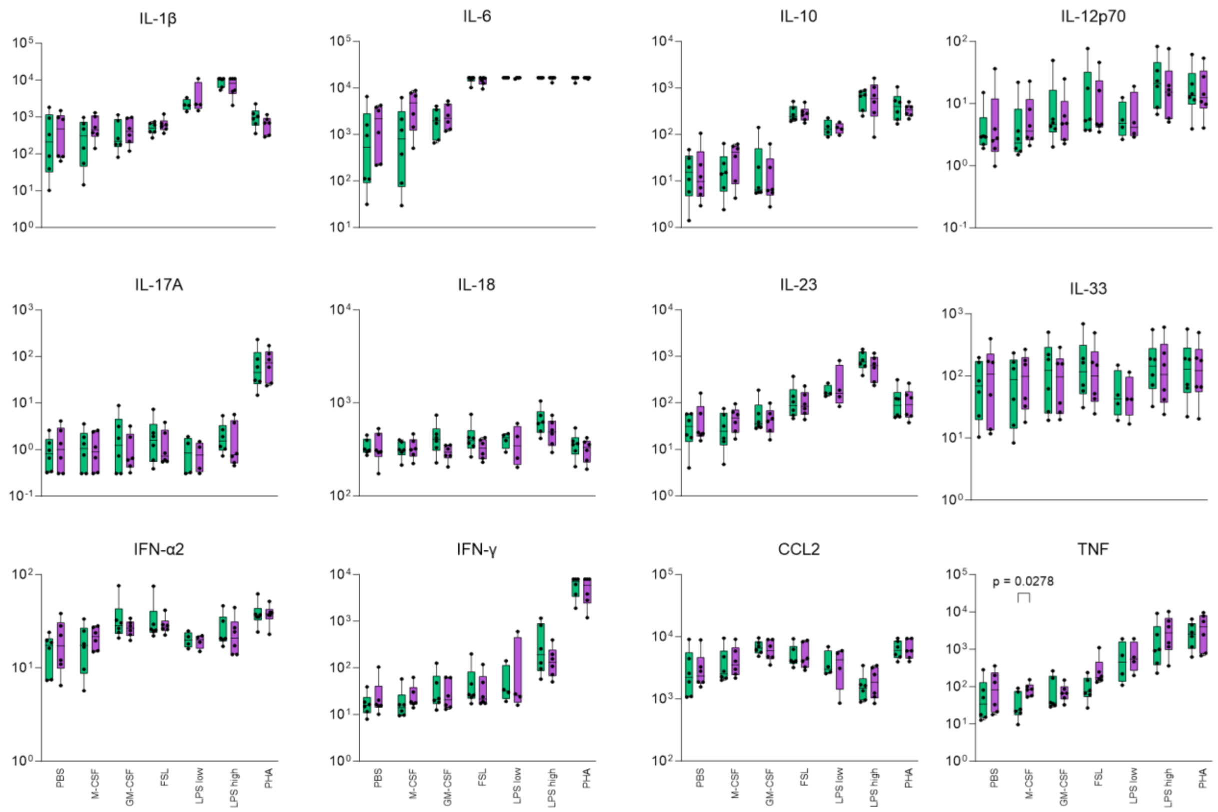

Fig.S5 Multiple cytokine concentrations before and after 1 month of azithromycin therapy

Blood samples were collected using the TruCulture™ system from sarcoidosis patients. Samples were taken at baseline (green) and after 1 month (blue) on azithromycin therapy. Blood was stimulated with null control (top row, n=10) or 100ng/ml LPS (bottom 3 rows, n=9) for 24h. Supernatants were analyzed for 13 cytokines by ELISA. Cytokines with results below the lower limit of detection of the assay are not shown. Boxplots show median (horizontal line), 95% confidence interval (box), and range (whiskers). Individual patient data are plotted as dots. One patient on low dose prednisolone therapy is plotted in orange. Data were analyzed using a linear mixed effects model. p values were corrected for multiple comparisons. Statistically significant results are shown on the plot.

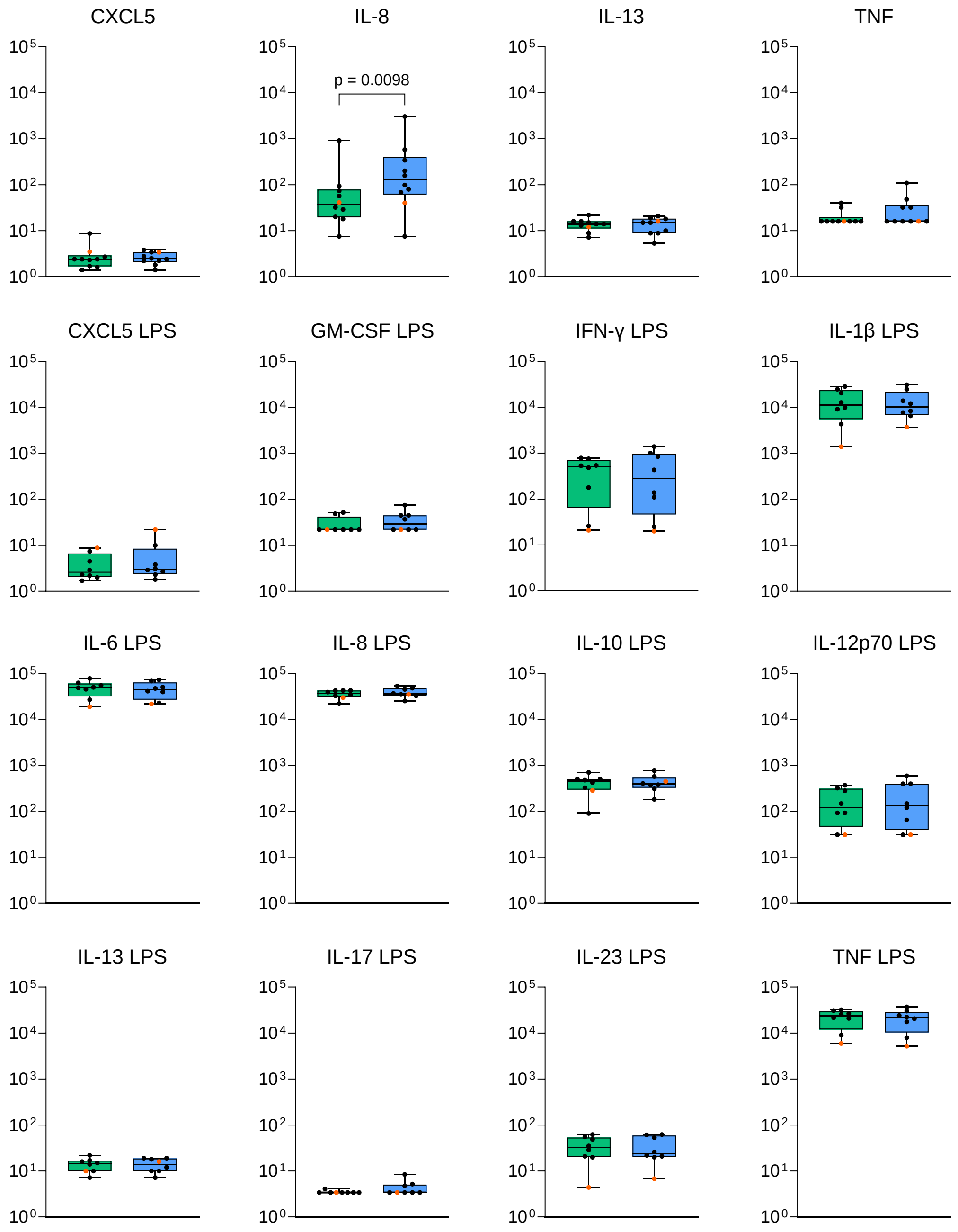

Fig.S6 Cell activation and regulatory markers and mTOR in sarcoidosis patients taking azithromycin

Blood samples were taken at baseline (green) and following 1 month (blue) and 3 months (purple) of azithromycin therapy. Individual patient data are plotted as dots (n=21). Patients taking oral corticosteroid therapy are plotted in orange. Blood monocytes and lymphocytes were analyzed for surface activation markers CD25 and CD11b, regulatory molecules CD200L and CD200R, and intracellular mTOR (mTORC1) activity. Data were analyzed using a linear mixed effects model. p values were corrected for multiple comparisons.

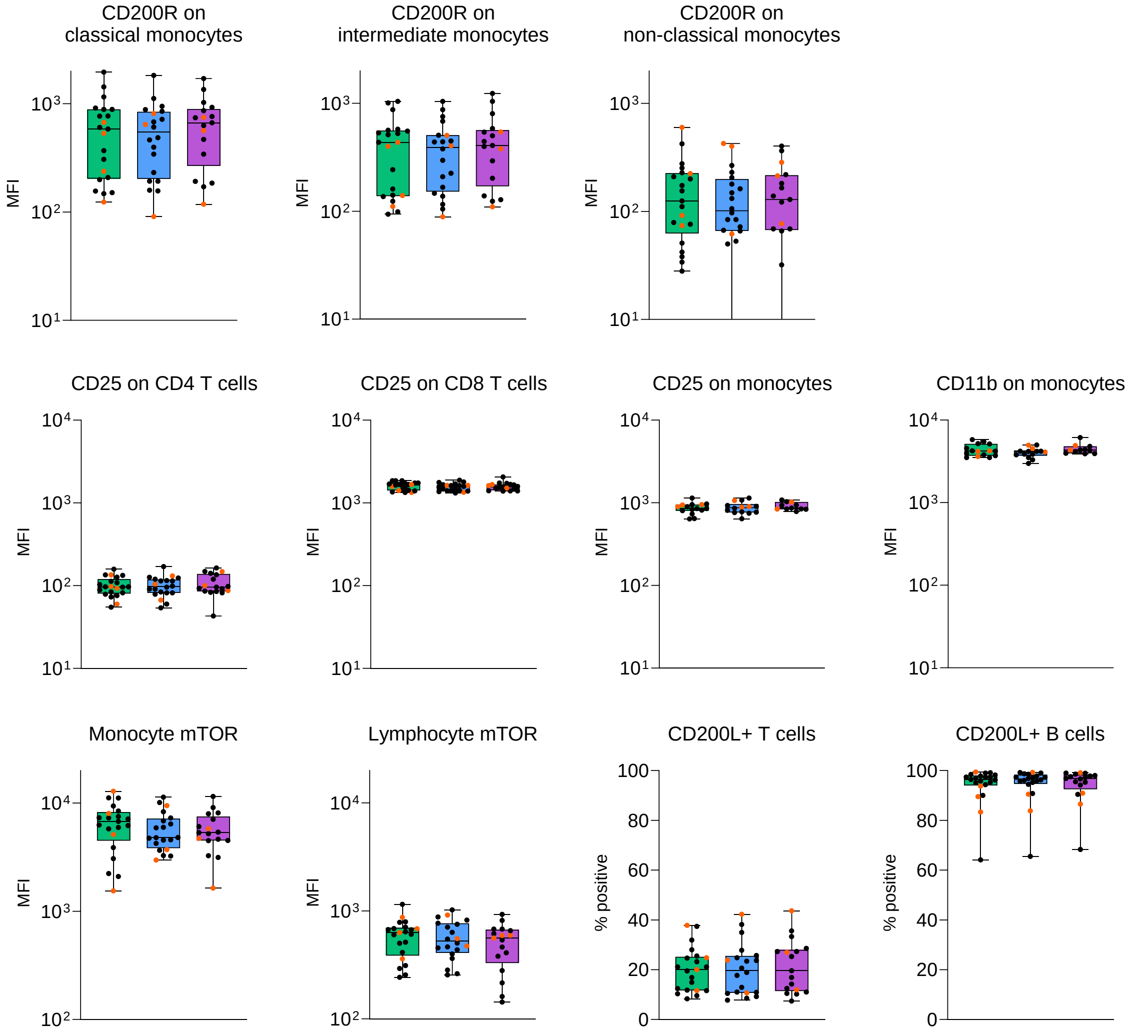

Table S1. Genes significantly upregulated and downregulated in sarcoidosis compared with controls

	Upregulated			Downregulated	
	Log2 fold change	-Log10 (BH.p-value)		Log2 fold change	-Log10 (BH.p-value)
ALAS2	3.6	4.25	***AGER***	-3.73	5.42
GMPR	3.14	3.66	***CTPS1***	-2.75	3.40
FCGR1A	2.67	2.22	***FAM129C***	-2.25	3.95
SERPING1	2.62	2.49	***PLCG1***	-2.22	4.30
NUDT1	2.46	5.06	***MTOR***	-2.12	5.42
TYROBP	2.41	6.66	***ZAP70***	-2.05	5.27
UBA52	2.3	4.85	***ITPR3***	-1.96	3.81
S100A9	2.25	5.16	***TYK2***	-1.89	4.37
IFITM1	2.24	5.91	***TRAF1***	-1.87	5.87
IFITM3	2.21	3.27	***CIITA***	-1.87	3.91
IDO1	2.19	2.95	***TLR9***	-1.85	3.15
IFIT1	2.17	2.95	***IKBKE***	-1.81	5.31
ANXA1	2.14	7.51	***FES***	-1.79	2.82
ATP6V0C	2.14	4.28	***CTLA4***	-1.72	3.48
S100A8	2.13	4.54	***FAM30A***	-1.72	3.27
HIST1H2AE	2.05	8.63	***ELAVL1***	-1.72	2.84
ISG15	2.02	3.20	***DDIT4***	-1.71	4.20
RIPK2	1.97	4.62	***ID1***	-1.71	3.65
LY96	1.96	5.45	***ICOS***	-1.68	2.50
IFI27	1.95	2.60	***FCRL2***	-1.67	2.09
CCR1	1.93	5.11	***LAT***	-1.66	5.20
RELB	1.9	4.29	***IL27RA***	-1.64	2.53
GBP5	1.9	2.50	***NEO1***	-1.6	2.00
OASL	1.8	4.16	***KMT2A***	-1.59	3.73
IFIT3	1.8	2.61	***TELO2***	-1.56	3.02
MSRB2	1.75	3.61	***STAT6***	-1.55	5.00
HLA-DRA	1.73	5.26	***CARD11***	-1.54	4.40
PRDX2	1.7	3.44	***RRAS2***	-1.54	3.46
TCL1A	1.7	2.36	***CEACAM3***	-1.53	4.21
NPRL3	1.67	2.79	***DNMT3A***	-1.51	5.42
IFI30	1.66	5.27	***MAP4K2***	-1.51	5.06
SERPINA1	1.66	4.82	***CCR7***	-1.5	4.79
TXN	1.65	4.98	***IRF4***	-1.48	4.30
BCL2A1	1.65	3.40	***NLRP1***	-1.45	5.91
HIST1H4E	1.58	5.87	***SIGIRR***	-1.43	2.67
UBE2L3	1.58	5.06	***IRF3***	-1.43	2.42
CGAS	1.56	2.50	***NOD2***	-1.43	2.06
PSMB8	1.54	4.95	***GHDC***	-1.41	2.14
S100A12	1.51	4.74	***BCL2***	-1.39	3.09

HLA-DPA1	1.51	4.63	***TRIM35***	-1.33	2.67
PSMB9	1.44	4.29	***TNFRSF1B***	-1.32	5.37
CD14	1.44	4.10	***POU2F2***	-1.32	3.90
HLA-DMB	1.43	3.46	***ITK***	-1.32	2.79
IFNGR1	1.42	4.62	***CD40LG***	-1.31	2.24
CTSA	1.41	5.06	***IKBKB***	-1.3	4.65
LYN	1.39	5.34	***IL21R***	-1.3	3.87
GBP2	1.39	3.89	***TNFRSF14***	-1.29	3.20
HIST1H2BD	1.38	2.14	***CTNND1***	-1.29	2.06
GSTP1	1.34	4.95	***NFATC2***	-1.27	3.79
RSAD2	1.33	2.12	***MDM2***	-1.24	4.77
NFE2L2	1.31	5.28	***SMAD3***	-1.24	4.30
TANK	1.29	5.16	***CD27***	-1.23	2.94
AP2M1	1.29	3.73	***ARHGEF1***	-1.21	4.10
CTSC	1.28	4.74	***DNMT1***	-1.2	2.38
CD52	1.28	4.51	***RNF126***	-1.2	2.26
CLEC4E	1.28	3.37	***S1PR4***	-1.18	3.15
PLEKHB2	1.27	5.91	***PTGER4***	-1.18	2.52
STAT1	1.27	2.27	***IL6ST***	-1.14	3.10
NRBF2	1.26	5.27	***MPZL1***	-1.14	2.14
CXCL5	1.26	2.05	***PANX1***	-1.13	2.33
TNFSF10	1.24	3.46	***SOCS5***	-1.12	3.35
ATF2	1.23	4.94	***TAB1***	-1.12	2.92
FPR1	1.19	2.24	***VAMP2***	-1.09	4.45
HIST1H4H	1.17	4.25	***GATA3***	-1.07	2.17
CD45R0	1.16	3.52	***TRIM26***	-1.06	3.07
HLA-DPB1	1.15	3.45	***ATM***	-1.04	3.05
CD46	1.14	4.75	***JAK3***	-1.04	2.66
YY1	1.13	5.28	***PRKCQ***	-1.03	3.47
PLAUR	1.13	3.65	***CD96***	-1.01	3.12
CCL5	1.13	2.47	***GRK6***	-1	5.20
IFNAR1	1.12	4.26	***CSF3R***	-1	3.71
RELA	1.11	2.63	***CREBBP***	-0.986	5.54
TNFSF13B	1.1	3.10	***IL4R***	-0.98	2.41
FCGR3A/B	1.08	3.10	***TRIM14***	-0.961	2.33
TBK1	1.07	3.09	***MAP3K14***	-0.93	3.35
GRB2	1.06	4.00	***NFE2L1***	-0.914	4.54
HIST1H2BF	1.05	5.42	***MYC***	-0.914	2.36
PLD3	1.05	3.43	***TSC2***	-0.881	2.48
HIST1H2BK	1.05	3.11	***IMPDH1***	-0.876	3.35
MYD88	1.03	3.89	***ACIN1***	-0.863	4.98
HIF1A	1.02	3.45	***HERC6***	-0.84	2.08
IRF2	1.01	5.45	***TMEM173***	-0.823	2.28
CCL4	1.01	2.02	***TRAT1***	-0.811	2.33
HIST1H2AC	0.998	4.40	***IKBKG***	-0.799	2.86
CXCL1	0.992	2.28	***ITGAX***	-0.774	2.58

CYBB	0.982	2.86	***ITGAL***	-0.763	3.06
AHR	0.979	2.27	***TCIRG1***	-0.741	2.78
RARRES3	0.958	3.00	***NPRL2***	-0.732	2.77
DTX3L	0.954	2.32	***SMARCA4***	-0.721	2.35
SLC7A5	0.941	2.23	***MTMR3***	-0.661	3.26
NFKBIA	0.932	3.73	***TNF***	-0.645	2.09
SGK1	0.931	2.60	***SMN1***	-0.641	2.17
CD86	0.922	2.05	***PIK3CD***	-0.609	4.16
SPI1	0.917	3.99	***EP300***	-0.608	2.33
CD163	0.916	2.49	***MAPK7***	-0.588	2.00
TRIM21	0.903	4.54			
CSF2RA	0.902	2.16			
HIST1H3H	0.9	4.75			
MAPK14	0.897	3.89			
CDC42	0.896	4.78			
LRR1	0.867	3.47			
MAP2K1	0.858	3.64			
PSMB10	0.854	2.53			
CD55	0.846	2.73			
XRCC6	0.844	3.95			
SP3	0.823	3.88			
CD81	0.806	2.53			
CFP	0.799	3.11			
AQP9	0.775	2.31			
MCL1	0.757	3.02			
UBE2S	0.723	2.94			
IL2RG	0.707	3.91			
SYK	0.691	2.82			
NPTN	0.685	3.17			
DYNLL2	0.679	3.77			
RHOA	0.675	2.19			
CD45RB	0.663	2.30			
STK26	0.647	2.75			

Appendix 1. Flow cytometry gating

Monocytes

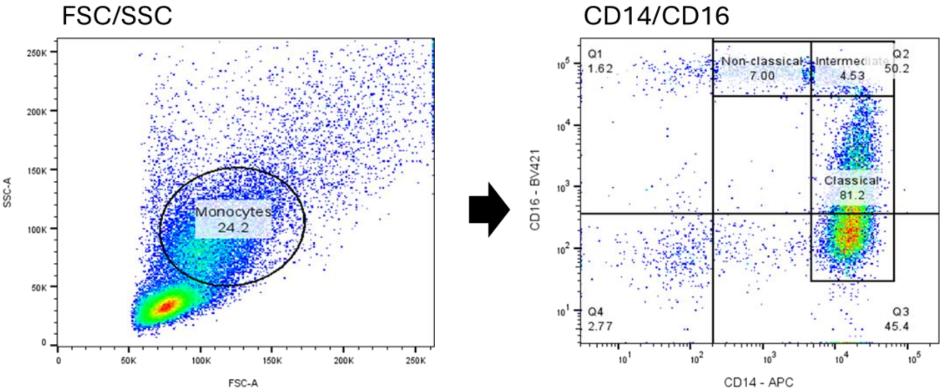

Lymphocytes

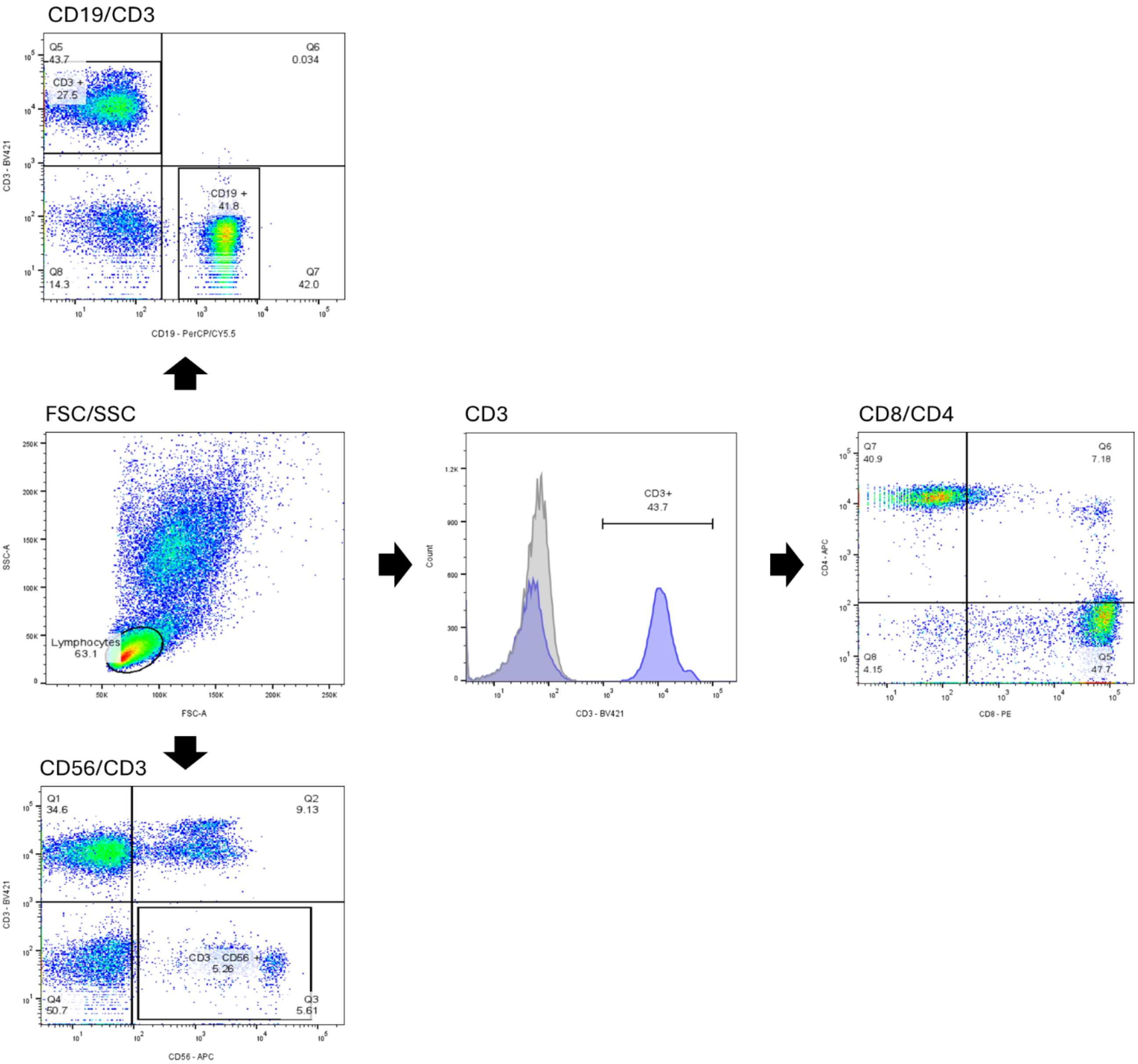

Supplement: Supplementary file 1 — Supplementary file1 (PDF 2111 KB) [file 408_2024_743_MOESM1_ESM.pdf]
